# A questionnaire-based survey on the diagnostic and therapeutic approaches for patients with STIC in Germany

**DOI:** 10.1007/s00404-023-06919-8

**Published:** 2023-01-27

**Authors:** Josche van der Ven, Valerie Catherine Linz, Katharina Anic, Mona Wanda Schmidt, Amelie Loewe, Slavomir Krajnak, Marcus Schmidt, Stefan Kommoss, Barbara Schmalfeldt, Jalid Sehouli, Annette Hasenburg, Marco Johannes Battista

**Affiliations:** 1 Department of Gynaecology and Obstetrics, University Medical Centre of the Johannes Gutenberg-University Mainz, Langenbeckstreet 1, 55131 Mainz, Rhineland-Palatinate Germany; 2 Department of Women’s Health, Tübingen University Hospital, Calwerstreet 7, 72076 Tübingen, Baden-Württemberg Germany; 3 Department of Gynaecology, University Medical Centre Hamburg-Eppendorf, Martinistreet 52, 20251 Hamburg, Germany; 4 Department of Gynaecology and Centre of Oncological Surgery, Charité Universitaetsmedizin Berlin Charité Campus Virchow-Klinikum, Augustenburger Platz 1, 13353 Berlin, Germany

**Keywords:** Serous tubal intraepithelial carcinoma, STIC, Ovarian cancer, Survey, SEE-FIM, Salpingectomy, BRCA

## Abstract

**Purpose:**

Despite the growing understanding of the carcinogenesis of pelvic high-grade serous carcinoma (HGSC) of the ovary and peritoneum and its precursor lesion serous tubal intraepithelial carcinoma (STIC), evidence-based proven recommendations on the clinical management of patients with STIC are lacking so far.

**Methods:**

A questionnaire containing 21 questions was developed to explore the clinical experience with patients with the diagnosis of STICs and the diagnostic, surgical and histopathological approaches in Germany. Overall, 540 clinical heads of department in all German gynaecological centres were asked to participate.

**Results:**

131 questionnaires (response rate 24.3%) were included in this survey. 45.8% of the respondents had treated one to three STIC patients during their career. 75.6% of the respondents performed opportunistic bilateral salpingectomies during other gynaecological surgeries. Most of the participants (31.3%) started with the SEE-FIM (Sectioning and Extensively Examining the FIMbria) protocol in 2014. It was requested by 39.7% centres for prophylactic salpingectomies, by 13.7% for both prophylactic and opportunistic salpingectomies and by 22.1% for neither of both. 38.2%, 1.5% and 24.4% of the participants would use the laparoscopic, transverse and midline laparotomic approach for a surgical staging procedure, respectively. 25.6% (54.7%) of the respondents recommended a hysterectomy in premenopausal (versus postmenopausal) patients with a STIC, 24.4% (88.4%) a bilateral oophorectomy and 50.0% (4.7%) an affected side oophorectomy (all *p* values < 0.001). Omentectomy, pelvic and para-aortic lymphadenectomy would be performed by 60.5% (64.0%), 9.3% (11.6%) and 9.3% (11.6%) of respondents in premenopausal (versus postmenopausal) patients (all *p* values > 0.05).

**Conclusion:**

Our survey highlights significant inconsistency in the management of patients with STIC. Prospective data are urgently needed to elucidate the clinical impact of a STIC lesion and its clinical management.

**Supplementary Information:**

The online version contains supplementary material available at 10.1007/s00404-023-06919-8.

## What does this study add to the clinical work

**Table:** 

Significant inconsistency in the management of patients with STIC resides among German gynaecological oncologists. Prospective data are urgently needed to clarify the impact of a STIC lesion and its clinical management.	

## Introduction

Epithelial ovarian cancer (EOC) is the most lethal gynaecological cancer with a 5-year survival rate of 49% [1]. High-grade serous ovarian carcinoma represents the most common histologic type of EOC and the majority of this cancer originate from precursor lesions of the fallopian tube. The latter include epithelia with a p53 signature, secretory cell outgrowths (SCOUT) as well as serous tubal intraepithelial carcinoma (STIC) [2]. STIC is also considered as the precursor lesion of other pelvic (i.e. peritoneal) high-grade serous carcinoma (HGSC) [3–6].

The lifetime risk for EOC is less than 2% in the general population. In contrast, women with proven *BRCA* germline mutations have an increased risk for developing ovarian cancer. The cumulative ovarian cancer risk up to the age of 80 years is 44% (95% CI 36%–53%) for *BRCA1* and 17% (95% CI 11%–25%) for *BRCA2* mutation carriers [7]. For these women, risk-reducing salpingo-oophorectomy (RRSO) is recommended and presents the most effective method of prevention so far [8, 9]. STIC and/or occult carcinoma is detected in approximately 10–15% of these cases [3], and isolated STIC in approximately 2% [10]. In contrast, the incidence of STIC in patients without familial predisposition of EOC is uncertain. A Canadian study reports STIC in 8 out of 9392 women (< 0.01%) with benign diagnoses [11]. Accordingly, a recent published Canadian population-based, retrospective cohort study of patients who underwent opportunistic salpingectomy or a control surgery (hysterectomy alone or tubal ligation) shows that the removal of the fallopian tubes in women at baseline risk for ovarian cancer reduces the risk for EOC [12]. In the future, opportunistic salpingectomies will likely increase in routine surgery as a strategy for EOC prevention.

The precursor lesion STIC is mostly located in the fimbriated end of the fallopian tube and typically demonstrates increased mitotic activity, significant atypia, architectural alterations and aberrant staining pattern for p53 (strong and homogenous, complete loss or cytoplasmic), which indicates the presence of a pathogenic mutation in p53[13]. The pathological work-up is clearly defined and should include the standardized SEE-FIM (Sectioning and Extensively Examining the FIMbria) protocol. The SEE-FIM protocol helps pathologists to better detect these STIC lesions and is nowadays established for RRSO after its first publication in February 2006 [14].

Women with a proven isolated STIC lesion are at substantial risk of developing HGSC. The 5- and 10-year risks to develop peritoneal carcinomatosis after a STIC diagnosis at RRSO are 10.5% (95% CI 6.2–17.2) and 27.5% (95% CI 15.6–43.9) [15], respectively, and predominantly in *BRCA1* mutation carriers [16, 17]. The corresponding risks for women without STIC at RRSO are 0.3% (95% CI 0.2–0.6) and 0.9% (95% CI 0.6–1.4) [15].

Besides being a precursor lesion, STICs may already be an indicator for an actually active HGSC of the ovary. Patients with incidental STIC in a low-risk population underwent surgery and three out of seven patients have been upstaged to an HGSC of the ovaries [18]. Therefore, German guidelines recommend informing a patient with a diagnosed STIC lesion about the risk of an already ongoing malignant process and discussing the possibility of surgical staging procedures [19]. However, no further specifications about the extent of the surgery, the methods of pre-surgical diagnostics or imaging are recommended due to the lack of data.

Once a STIC is diagnosed, no established clinical management regarding diagnostics and treatment is available for these patients. Our survey investigates how STIC is diagnosed and treated in gynaecological centres in Germany to critically discuss the actual management.

## Materials and methods

A questionnaire with 21 multiple-choice questions was intraprofessionally developed to investigate the management and experience with STIC patients among German gynaecological centres (see supplementary). The online questionnaire was generated using SoSci Survey and was made available to users via www.soscisurvey.de, an open source platform for non-profit research [20]. After the approval of the scientific board of the German working group for ovarian cancer (Arbeitsgemeinschaft Gynäkologische Onkologie (AGO), Organkomission OVAR), a link to the questionnaire was sent to 540 email addresses from all available German gynaecological centres in February 2020. Two reminders were sent in March and April 2020. The list of all German gynaecological centres was provided by the German Society of Gynaecology and Obstetrics (DGGG) in 2006, subsequently updated and used previously for other questionnaire-based analyses. The questionnaire was in German, translated into English and is shown in the supplementary.

### Items

Questions included general data concerning the hospitals’ organisational structures and their specific, tumour-related data.

At first, questions comprised the hospitals’ characteristics including, e.g. the number of beds or whether these centres were teaching or university hospitals. Furthermore, information concerning the professional training in gynaecological oncology was collected as well as the numbers of cancer patients treated in each centre (see supplementary, general information).

Detailed information about the pathological analysis and the usage of the standardized SEE-FIM protocol—referred to as ultrastaging in the questionnaire—was obtained subsequently (see supplementary, histological handling). STIC-related data, such as the number of patients diagnosed with a STIC, were gathered. In addition, we created hypothetical questions concerning diagnostics and individual treatment decisions for STIC patients (see supplementary).

These questions included diagnostic procedures, access to surgery, extent of surgery in pre-and postmenopausal women as well as adjuvant chemotherapy.

### Statistical analysis

Raw data were obtained through SoSci Survey and listed in Excel files. Data were analysed using SPSS 26.0 (IBM Corp., Armonk, NY, USA) and summarized as means (± standard deviation) or proportions (%). Chi-square test was used to assess significant differences between proportions after exclusion of not available (n/a) answers. A two-sided *p* value of < 0.05 was considered statistically significant.

## Results

### Experience of cancer centres

Overall, 131 questionnaires were completed sufficiently to be included within the final statistical analysis (24.3%). Within these 131 returned questionnaires, 75.1% of the questions were answered. Hospitals were categorized in three types. 14.5% of the institutions were university hospitals, 72.5% teaching hospitals and 13% hospitals with no additional designation (Table 1). Most of the participating hospitals (27.5%) possessed more than 60 beds in their department (Table 1). 51.9% were members of the AGO (Table 2), whilst 48.9% of all centres were certified as gynaecological oncology centres and 63.4% were certified as breast cancer centres in accordance with the German cancer society (Deutsche Krebsgesellschaft). Most of the centres treated 201–250 breast cancer patients and 13–24 ovarian cancer patients per year. 27.5% of all participants had at least one physician officially specialized and certified in gynaecological oncology working in their department (Table 2).

**Table 1:** Characteristics of the participating hospitals

	*n* (%)
General data	
Invitations sent	540 (100)
Respondents	131 (24.3)
Type of hospital	
University hospital	19 (14.5)
Teaching hospital	95 (72.5)
No additional designation	17 (13.0)
Number of beds	
< 10	1 (0.8)
0–20	6 (4.6)
1–30	24 (18.3)
31–40	29 (22.1)
41–50	20 (15.3)
51–60	14 (10.7)
> 60	36 (27.5)
n/a	1 (0.8)
Certified oncological centre	
Gynaecological cancer centre^1^	64 (48.9)
Breast cancer centre^1^	83 (63.4)
Number of treated breast cancer patients per year	
< 100	17 (13.0)
100–150	13 (9.9)
151–200	10 (7.6)
201–250	19 (14.5)
251–300	10 (7.6)
301–350	2 (1.5)
> 350	13 (9.9)
n/a	47 (35.9)
Number of treated ovarian cancer patients per year	
0–6	5 (3.8)
7–12	31 (23.7)
13–24	38 (29.0)
> 24	31 (23.7)
n/a	26 (19.8)
Number of treated STIC patients so far	
1–3	60 (45.8)
4–9	14 (10.7)
> 9	6 (4.6)
None	18 (13.7)
n/a	33 (25.2)

*n/a* not available, *STIC* serous tubal intraepithelial carcinoma

^1^In accordance with the German cancer society (Deutsche Krebshilfe, DKG)

**Table 2:** Information regarding the experience and training in gynaecological oncology

	*n* (%)
Number of specialists trained in gynaecological oncology	
1	36 (27.5)
2	26 (19.8)
3–4	28 (21.4)
> 5	8 (6.1)
n/a	33 (25.2)
Experiences in gynaecological oncology in years	
< 4	5 (3.8)
4–10	11 (8.4)
10	89 (67.9)
n/a	26 (19.8)
Affiliation to organizations/task forces	
Member of the AGO^1^	68 (51.9)
n/a	23 (17.6)
Usage of German S3 guideline of ovarian cancer	
Never	5 (3.8)
1–3x	39 (29.8)
4–9x	38 (29.0)
> 9x	23 (17.6)
n/a	26 (19.8)
Internal STIC workshop/training	
Yes	65 (49.6)
No	39 (29.8)
n/a	27 (20.6)

*n/a* not available, *STIC* serous tubal intraepithelial carcinoma

^1^German working group for gynaecological oncology (*AGO* Arbeitsgemeinschaft Gynäkologische Onkologie)

### Personal experience of participants

Participants reported long experiences in gynaecological oncology (67.9% more than 10 years, Table 2). 45.8% had treated one to three STIC patients so far (Table 1). Nearly half of all participants (49.6%) had already discussed the topic STIC as part of a workshop in their department (Table 2). The participants regularly used the German evidence-based (S3) guideline for ovarian cancer in their clinical routine within the last year [19] (Table 2). Most centres (75.6%) performed opportunistic salpingectomies; nearly a third of the respondents started with those in 2014 (31.3%).

### Diagnostics

Most of the centres implemented the SEE-FIM protocol in 2014 (31.3%). 13.7% applied the protocol for all salpingectomies, 39.7% only for prophylactic salpingectomies and 22.1% for neither.

**Fig. 1:**
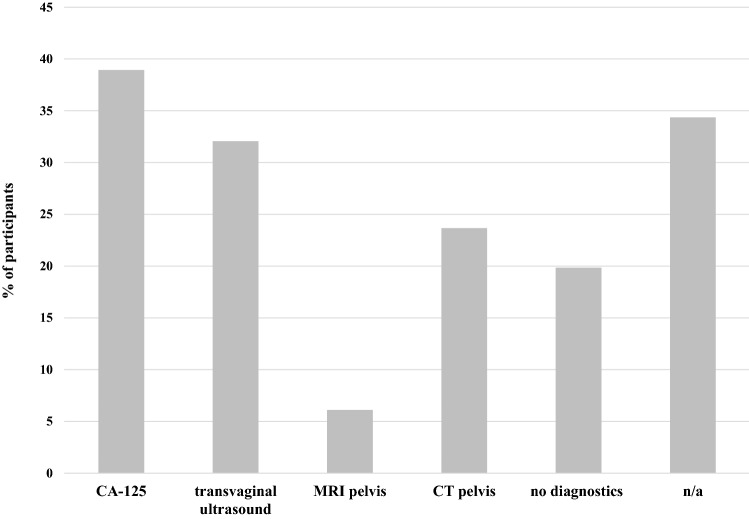
Diagnostics performed in case of an isolated STIC. Multiple answers were possible. *CT* computed tomography, *n/a* not available, *MRI* magnetic resonance imaging computed tomography, *STIC* serous tubal intraepithelial carcinoma

The participants were asked hypothetically which diagnostic procedures they would perform in case of an isolated STIC lesion. The options included serum CA-125 (38.9%), transvaginal ultrasound (32.1%), magnetic resonance imaging (MRI) of the pelvis (6.1%), computed tomography (CT) of the pelvis (23.7%) and no diagnostics at all (19.8%) (see Fig. 1).

### Treatment

**Fig. 2:**
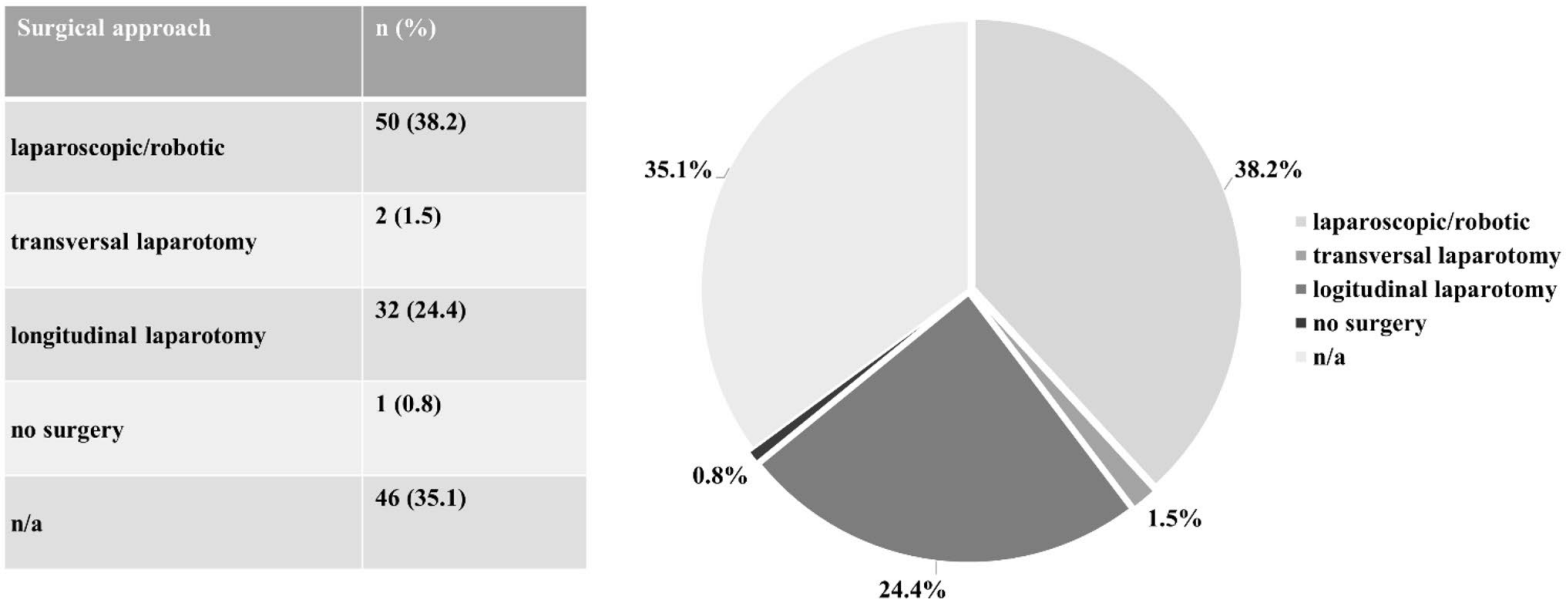
Surgical approach for isolated STIC. *n/a* not available, *STIC* serous tubal intraepithelial carcinoma

With regard to the therapeutical approach, most surgeons would choose a robotic/laparoscopic approach to perform a surgical staging procedure (38.2%), see Fig. 2.

**Fig. 3:**
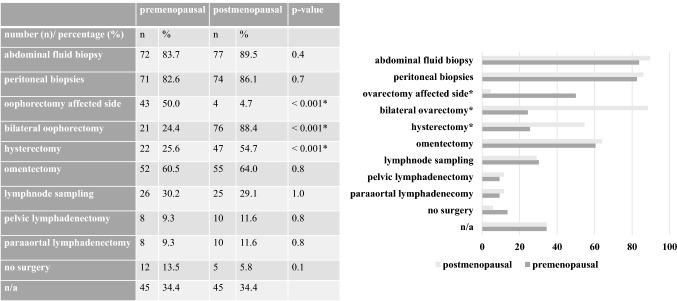
Comparison of hypothetical surgical procedures in pre- versus postmenopausal STIC patients. * = significant results *p* < 0.001, multiple answers were possible. *n/a* not available; n/a were excluded in the analysis. *STIC* serous tubal intraepithelial carcinoma

In premenopausal (postmenopausal) STIC patients, 25.6% (54.7%, *p* < 0.001) of the centres stated to perform a hysterectomy, 24.4% (88.4%, *p* < 0.001) a bilateral oophorectomy, 50.0% (4.7%, *p* < 0.001) an affected side oophorectomy. Omentectomy, pelvic and para-aortic lymphadenectomy would be performed by 60.5% (64.0%), 9.3% (11.6%) and 9.3% (11.6%) centres in premenopausal (postmenopausal) STIC patients (all *p* values > 0.05), respectively, see Fig. 3. Very few participants opted to treat a STIC patient with adjuvant chemotherapy (2.3%).

## Discussion

Our survey highlights many inconsistencies in the management of patients with STIC among gynaecological departments in Germany. Even though 49.6% of the centres had a STIC-related training held at their centre, most clinicians had only treated very few patients during their medical career.

A relevant number of participants do not perform an opportunistic salpingectomy routinely during everyday surgery, neither do all of them request the correct pathological examination in accordance with the SEE-FIM protocol, especially for high-risk patients. According to the ESMO–ESGO Consensus, the SEE-FIM protocol should be performed in all risk-reducing prophylactic surgery specimens [21].

Diagnosing STIC is challenging and shows only moderate reproducibility. Therefore, a recently published systematic review suggests not only the use of the SEE-FIM protocol, but also evaluation by a subspecialized pathologist, rational use of immunohistochemical staining, and obtaining a second opinion from a colleague to secure the diagnosis [22].

Recent literature still mostly provides case series of STIC patients with individual diagnostic approaches [15, 23, 24]. Similarly, our findings showed a great variety of approaches as well. Regarding diagnostic procedures, most of the participants would perform a transvaginal ultrasound and control serum CA-125. To date, no effective screening tool exists to monitor STIC patients [25]. Most of the published studies include annual clinical checkups with pelvic ultrasound and in some cases routine evaluation of serum CA-125[17]. *BRCA* status should additionally be checked in cases of isolated STIC. However, in general, no routine screening for ovarian HGSC should be offered to women of the general population [26, 27].

Peritoneal restaging should be considered in cases of incidentally detected, apparently isolated STIC lesions [21]. A systematic review of the literature in 2018 suggests that a staging procedure as an additional treatment after RRSO and the diagnosis of an isolated STIC is associated with a lower risk of recurrence [24]. A surgical staging for patients with STIC mostly included hysterectomy, omentectomy, pelvic and para-aortic lymph node dissection and peritoneal washing [24]. Interestingly, the routine use of peritoneal biopsies during RRSO does not seem to improve the detection of occult malignancies [28].

Chay et al. suggest that a complete staging surgery should be considered for non-*BRCA* patients with a STIC lesion as well, since in three out of seven STIC cases, staging surgery led to an upstaging from STIC to HGSC of the ovaries [18]. 38.2% of the centres in our survey would advise a laparoscopic surgery after the diagnosis of STIC, even though guidelines for ovarian cancer recommend a laparotomy for surgical staging. However, data concerning the best approach for the surgical staging in patients with STIC are lacking so far. A systematic Cochrane review could not help to quantify the value of laparoscopy for the management of early stage ovarian cancer as routine clinical practice [19, 29]. Furthermore, a cohort study of the AGO OVAR regarding ovarian borderline tumours could not show any significant impact of the initial surgical approach on recurrence either [30].

Remarkably, the completeness of surgical staging in patients with early ovarian cancer is significantly associated with better outcomes compared to incomplete surgical staging procedures [31]. This association has not been proven for patients with a STIC lesion. We found significant differences in strategies regarding the extent of surgical procedures in pre- versus postmenopausal women after the diagnosis of STIC. Most centres would perform oophorectomies for the affected side only in premenopausal women, while a bilateral oophorectomy would be performed in postmenopausal women. In view of these results, the questionnaire might be modified in the future and stratify the women by the status of the family planning rather than their menopausal status. No data are yet available concerning STIC patients and the effects of delayed oophorectomy to prevent early onset of menopause and non-cancer-related morbidity, but these questions are currently being addressed by an ongoing clinical trial (ClinicalTrials.gov identifier: NCT04294927, ISRCTN 25,173,360, and ClinicalTrials.gov identifier: NCT04251052).

Patrono and colleagues reviewed 78 STIC cases, of whom 16.4% received adjuvant chemotherapy [23]. In our survey, adjuvant chemotherapy was rarely recommended by the centres. In general, adjuvant chemotherapy for intraepithelial neoplasia with negative washing is not advised any longer [21].

Routine surveillance for every patient with STIC is recommended for the next years, because the time from STIC to invasive cancer has been suggested to be approximately 7 years and has guided the recommendation for RRSO at 35–40 years of age in *BRCA1* patients [32].

To the best of our knowledge, we performed the first survey regarding the clinical management of patients with STIC in Germany so far. The response rate to our study was moderate but within the range of health care professionals’ surveys [33]. Unfortunately, many of the participants only finished the first part of the survey and did not take part in the more detailed case-related questions. A further limitation is the low experience of the centres with STIC patients, since most of the respondents had treated only one to three STIC cases up to date due to its low incidence. Furthermore, a questionnaire with hypothetical questions should be interpreted with caution because clinical decision in the real world might be different. The questionnaire just comprised 21 questions and some important questions were not asked, e.g. if only gynaecological pathologists performed the histological examination. It remains unclear if the centres have their own clinical standard with a predefined protocol concerning diagnostics and treatment of STIC. Therefore, future studies should be performed to update the clinical day routine prospectively.

Our survey demonstrates the lack of consistency in the management of patients with STIC in Germany. It underlines the need for more information about isolated STIC, especially regarding the best approach and extent of the surgical staging as well as the risk for isolated lymph node metastasis without peritoneal carcinomatosis, in general. A prospective register collecting clinical data of STIC patients might be helpful to evaluate the clinical courses of the disease and to identify important diagnostic and therapeutic tools. This may establish an evidence-based strategy and will finally lead to a validated guideline for diagnostic procedures and treatment of women with a STIC to support gynaecological oncologists in their daily practice. Additionally, interdisciplinary educational programmes should be established to increase the awareness.

## Data availability statement

All data generated or analysed during this study are included in this article and its tables and figures. Further enquiries can be directed to the corresponding author.

## Supplementary Information

Below is the link to the electronic supplementary material.

Supplementary file1 (DOCX 39 kb)
