# Supplementary material for: A questionnaire-based survey on the diagnostic and therapeutic approaches for patients with STIC in Germany
Source: Arch Gynecol Obstet. 2023 Jan 27;308(2):527–34. doi: 10.1007/s00404-023-06919-8 (PMC10293341; doi:10.1007/s00404-023-06919-8)
Supplement: Supplementary file 1 — Supplementary file1 (DOCX 39 kb) [file 404_2023_6919_MOESM1_ESM.docx]

## Supplementary

## A questionnaire based survey on the diagnostic and therapeutic approaches for patients with STIC in Germany

*Josche van der Ven^1^*^†^*, Valerie Catherine Linz^1^*^†^*, Katharina Anic^1^, Mona Wanda Schmidt^1^, Amelie Loewe^1^, Slavomir Krajnak^1^, Marcus Schmidt^1^, Stefan Kommoss^2^, Barbara Schmalfeldt^3^, Jalid Sehouli^4^, Annette Hasenburg^1^, Marco Johannes Battista^1^*

1 Department of Gynaecology and Obstetrics, University Medical Centre of the Johannes Gutenberg-University Mainz, Mainz, Langenbeckstreet 1, 55131 Mainz, Rhineland-Palatinate, Germany.

2 Department of Women's Health, Tübingen University Hospital, Calwerstreet 7, 72076 Tübingen, Baden-Württemberg, Germany.

3 Department of Gynaecology, University Medical Centre Hamburg-Eppendorf,

Martinistreet 52, 20251 Hamburg, Hamburg, Germany.

4 Department of Gynaecology and Centre of Oncological Surgery, Charité Universitaetsmedizin Berlin Charité Campus Virchow-Klinikum, Augustenburger Platz 1,

13353 Berlin, Berlin, Germany.

^†^These authors have contributed equally to this work and share first authorship.

**Corresponding author:**

Dr. med. Valerie Catherine Linz

Department of Gynaecology and Obstetrics,

University Medical Centre of the Johannes Gutenberg-University Mainz,

Langenbeckstreet 1,

55131 Mainz,

Germany.

Phone: 0049 6131 17 0

Fax: 0049 6131 17 3415

E-Mail: [valerie.linz@unimedizin-mainz.de](mailto:valerie.linz@unimedizin-mainz.de)

ORCID Number 0000-0002-8018-5484

Questionnaire translated into English

***General data***

1. *Are you a member of the AGO (Arbeitsgmeinschaft Gynäkologische Onkology; German gynaecological oncology working group)?*

*Yes*

*No*

1. *How many beds do you have at your department?*

*<10*

*10-20*

*21-30*

*31-40*

*41-50*

*51-60*

*>60*

1. *Is your clinic a…*

*teaching hospital*

*University hospital*

*none of both (no designation)*

1. *Is your clinic a…*

*DKG certified gynaecological oncology center*

*DKG certified breast cancer center*

*None of both*

*Other*

1. *How many physicians in your department are specialized in gynaecological oncology?*

*1*

*2*

*3-4*

*>5?*

1. *How many years have you been working in gynaecolgocial oncology?*

*<4*

*4-10*

*10*

1. *How often did you use the German S3 manual ‘Diagnostic, therapy and aftercare for malignant ovarian tumors’ in your clinical routine last year?*

*Never*

*1-3x*

*4-9x*

*>9x*

1. *How many patients with newly diagnosed ovarian cancer are treated at your department per year?*

*0-6*

*7-12*

*13-24*

*>24*

1. *How many patients with newly diagnosed breast cancer are treated at your department per year?*

*<100*

*100-150*

*151-200*

*201-250*

*251-300*

*301-350*

*>350*

***Histology-related data***

1. *Have you discussed STIC at an internal workshop or training at your department so far?*

*yes*

*no*

1. *How many risk reducing bilateral salpingectomies do you perform per year at your department?*

*0-6*

*7-12*

*13-24*

*>24*

1. *Do you perform opportunistic bilateral salpingectomies at your department?*

*yes*

*no*

1. *At which timepoint have you established opportunistic bilateral salpingectomies at your department?*

*since this year (2020)*

*since 2017*

*since 2014*

*since 2011*

*before 2011*

1. *How many patients with an isolated STIC have you treated so far?*

*1-3*

*4.-9*

*>9*

*none*

1. *Do you perform ultra staging (SEE-FIM protocol) for…*

*all salpingectomies*

*prophylactic salpingectomies with high risk family gene or positive family history*

*no salpingectomies*

1. *If yes, since when do you perform ultra-staging (SEE-FIM protocol)*

*since this year (2020)*

*since 2017*

*since 2014*

*since 2011*

*before 2011*

*We do not perform ultra-staging.*

***STIC related case***

*From now on questions relate to the following scenario: You have performed a bilateral salpingectomy: Histological tests show an isolated STIC lesion (without hints of invasive cancer).*

*17. Which diagnostical measures would you take?*

*CA125*

*vaginal ultrasound*

*MRI pelvis*

*CT pelvis*

*none*

*18. Which surgical measures would you perform on a healthy premenopausal patient (without any mutation, up to ASA 3)?*

*abdominal fluid biopsy*

*peritoneal biopsy*

*oophorectomy affected side*

*bilateral oophorectomy*

*hysterectomy*

*omentectomy*

*lymphnode sampling*

*pelvic lymphadenectomy*

*paraaortal lymphadenectomy*

*no surgery*

*19. Which surgical measures would you perform on a healthy postmenopausal patient (without any mutation, up to ASA 3)?*

*abdominal fluid biopsy*

*peritoneal biopsy*

*oophorectomy affected side*

*bilateral oophorectomy*

*hysterectomy*

*omentectomy*

*lymphnode sampling*

*pelvic lymphadenectomy*

*paraaortal lymphadenectomy*

*no surgery*

*20. Which surgical approach would you choose?*

*laparoscopic/robotic*

*longitudinal laparotomy*

*transverse laparotomy*

*no surgery*

*21. Would you perform chemotherapy on a patient with isolated STIC?*

*yes*

*no*

ASA 3: **American Society of Anesthesiologists** Classification 3 (Patient with severe systemic disease)

CT: computed tomography

DKG (Deutsche Krebshilfe): German cancer society

MRI: magnetic resonance imaging

SEE-FIM: Sectioning and Extensively Examining the FIMbria

STIC: serous tubal intraepithelial carcinoma
